# A Comparison of Calcium Hydroxyapatite and Dextranomer/Hyaluronic Acid for the Endoscopic Treatment of Vesicoureteral Reflux

**DOI:** 10.1155/2013/263602

**Published:** 2013-10-23

**Authors:** Tin C. Ngo, Ilene Yi-Zhen Wong, William A. Kennedy

**Affiliations:** ^1^Department of Urology, Stanford University School of Medicine, 300 Pasteur Drive, S-287, Stanford, CA 94305, USA; ^2^Lucille Packard Children's Hospital, Stanford, CA 94305, USA

## Abstract

*Purpose*. Minimal data exists comparing dextranomer/hyaluronic acid (Dx/HA) and calcium hydroxyapatite (CaHA) for the endoscopic treatment of VUR in the hands of a single user. *Materials and Methods*. We reviewed our consecutive single-user case series of 27 children (42 ureters) receiving endoscopic treatment with CaHA and 21 children (33 ureters) who received Dx/HA injection. Children receiving CaHA injections were divided into two groups of 13 and 14 patients (Coaptite I and II) to assess the learning curve effects. Postoperatively, RBUS and VCUG were performed. Multiple regression analysis was performed to assess statistical significance of success rates. *Results*. The total CaHA group had a per-ureter success rate (Grade 0) of 52% after one injection. When separated into two cohorts, the single injection per-ureter success rates were 43% for Coaptite I and 62% for Coaptite II. In contrast, the Dx/HA series had a single injection per-ureter success rate (Grade 0) of 78%. *Conclusions*. Our consecutive case experience shows improved results for Dx/HA compared to CaHA, though the learning curve effects and evolution of injection technique likely played a role in the improved outcomes in the Dx/HA cohort. A randomized controlled multicenter trial would provide the most accurate data comparing these two agents.

## 1. Introduction

Recent years have seen a dramatic rise in the endoscopic treatment of vesicoureteral reflux (VUR) as an alternative to traditional ureteral reimplantation and antibiotic therapy [1, 2]. Prior to 2001, endoscopic therapy in the United States was limited to primarily off-label use of a variety of injectables, including polytetrafluoroethylene (Teflon) and polydimethylsiloxane (Macroplastique) which showed efficacy of >70% in clinical trials. They were later shown to have possible migration risks and never received FDA approval [3]. Subsequently, attention was turned toward larger-sized molecules (greater than 65 *μ*m in size), including calcium hydroxyapatite (CaHA, Coaptite) and dextranomer/hyaluronic acid (Dx/HA, Deflux). 

CaHA is a 100 *μ*m synthetic particle with a chemical composition identical to bone and teeth. The spherical molecules are delivered in an aqueous-based gel carrier. It has been widely used in the United States in dentistry, orthopedic surgery, plastic surgery, and ear, nose, and throat surgery. Coaptite was approved in 2005 for urologic use in the treatment of stress urinary incontinence [4]. Animal studies on calcium hydroxylapatite have shown it to be biocompatible with no associated migration risk. In contrast to Dx/HA, it is radio-opaque, allowing visualization of mound placement on plain films. 

Dx/HA remains the only injectable currently approved by the FDA for the treatment of VUR. CaHA is widely used in Europe for the treatment of VUR, but, in the United States, there have been limited data regarding CaHA. The largest series reporting on CaHA includes results from a 2-year multicenter trial that showed good durability of treatment but variable efficacy rates. This may be due to the procedural learning curves among the ten institutions involved [5]. At the present time, no US data exists comparing the results for CaHA versus Dx/HA in the hands of a single user and institution.

## 2. Materials and Methods

After the Institutional Review Board approval, a retrospective review was performed of our consecutive case series of 57 children undergoing endoscopic therapy for the treatment of VUR from 2001 to 2006. Children who did not have follow-up VCUG were excluded (*n* = 9).

Patients were selected for surgical intervention upon failure of resolution of reflux after at least one year of nonoperative management. It is our practice to delay any surgical treatment of VUR in patients who exhibit signs of dysfunctional voiding until it has been treated to resolution. We did not deviate from this practice in this study cohort. All injections were performed under a general anesthetic. The first 27 children (42 ureters) received CaHA under cystoscopic guidance. A 21-gauge needle was inserted 2-3 mm distal to the ureter (STING procedure) with injection of 0.4 to 1.6 mL of CaHA [6]. The procedural endpoint was visual occlusion of the ureteral meatus. The first 13 CaHA patients were treated as part of an FDA trial and received intraoperative on-table cystograms to identify passive reflux. On-table cystograms were not performed on any of the remaining patients in this study.

The subsequent 21 children (33 ureters) treated with Dx/HA were injected using the QMed proprietary 23-gauge endoscopic needle. The Hydrodistention Implantation Technique (HIT procedure) using intraureteric injection as described by Kirsch et al. was used on all patients undergoing treatment with Dx/HA [7]. This additional injection was not performed on the patients undergoing treatment with CaHA.

Renal bladder ultrasound and VCUG were performed 1 to 3 months after injection. Patients with persistent reflux were given the option of a second injection. A total of 41 patients received a single injection, while seven patients (5 CaHA and 2 Dx/HA) received a second injection. Complete resolution of reflux was classified as a Grade 0 Success. Grade 1 Success, on the other hand, was defined as those with improvement in their reflux to Grade 1 or better.

All data were summarized using descriptive statistics. Patient demographics including age, gender, grade and laterality of reflux, and presence of dysfunctional voiding were collected, and Student *t*-tests and Fisher's Exact test were performed to determine statistically significant differences between the Dx/HA and CaHA cohorts. Multivariable regression analysis was performed to determine the statistical significance between the treatment groups' efficacy rates, controlling for cohort, age, presence of dysfunctional voiding, gender and grade of reflux. 

## 3. Results

Table 1 lists the patient characteristics of the three cohorts. Significantly, more patients who were injected with CaHA had a history of dysfunctional voiding (9 in the Coaptite I cohort, 2 in the Coaptite II cohort), compared to no patients in the Dx/HA group. However, these behaviors were treated to resolution prior to undergoing endoscopic treatment of their VUR. Examination of our cohorts also shows that the Dx/HA cohort had a younger average age, higher male/female ratio, and a higher average grade of reflux than the total CaHA cohort.  Table 2 lists the distribution of the grade of reflux in our cohort. Most of the patients had grades II or III VUR.

The comparative success rates between our cohorts are shown in Table 3. When examined as a group, patients injected with CaHA had a Grade 0 single injection ureteral success rate of 52%. When separated into two cohorts to control for the learning curve, the Grade 0 single injection ureteral success rates were 43% for Coaptite I (“early”) and 62% for Coaptite II (“late”). After two injections, Grade 0 ureteral success rate for the CaHA group was 67% (62% for Coaptite I and 71% for Coaptite II). The Dx/HA group had a single injection ureteral success rate of 78%. 

Grade I ureteral success rate for the CaHA group was 80% after two injections (9 total ureters reinjected). In comparison, the Dx/HA group had an 88% Grade I cure rate after one injection (follow-up for the two Dx/HA patients that were reinjected is not available).

Multivariate analysis confirms that the difference between single injection Dx/HA and the CaHA success rates is statistically significant (*P* < 0.01), as is the difference between the success rates of the Dx/HA group and the Coaptite II group (*P* = 0.03).

## 4. Discussion

Since FDA approval in 2001 of Dx/HA for the endoscopic treatment of VUR, subtrigonal injection of biomaterials has become an increasingly prevalent first-line treatment for VUR in children [2]. At the present time in the United States, Dx/HA is the most commonly utilized biomaterial; however, centers in Europe and Canada have reported the successful use of a number of other materials, including polydimethylsiloxane and CaHA, with success rates ranging from 60 to 80%. An increasing body of literature suggests that different bioinjectables in the hands of a single user may have similar efficacy rates [8].

In 2008, Alkan et al. reported no significant difference between reflux resolution rates in a study of children injected with either Dx/HA (47 ureters) and CaHA (22 ureters) [9]. The literature concerning the use of CaHA for VUR in the United States consists of Mevorach et al's 2006 report on the results of a multicenter FDA trial of 98 patients [5]. Notably, this study recruited patients from the years 2001-2002, at a point before the widespread adoption of bioinjectables in the United States. The timing of this study is relevant to the given studies by Kirsch et al. showing the importance of the learning curve and technique modification on efficacy rates for endoscopic treatment of VUR. In this study, Mevorach et al. showed a global cure rate after two injections of 32% with a 46% ureteral cure rate after two injections. 55% of all patients received at least two injections. Subset analysis of the primary center's experience with 35 patients showed improved statistics including a 66% patient and 72% ureteral cure rate. It should be noted that these numbers for the primary center are after phase 2 and 3 safety trials and likely represent a point further along on the learning curve.

The primary center success rate reported by Mevorach et al. is similar to the European single-center studies of CaHA, which have shown 70–80% success rates after two injections [8, 10, 11]. 

One of the most important observations to come out of the literature concerning Dx/HA is the steep learning curve for injectables. Kirsch et al. showed a dramatic improvement after the first 20–30 cases in their two-surgeon experience, suggesting a 10–15 case learning curve per surgeon [12]. Therefore, we chose to divide our total CaHA group into two cohorts of 13-14 cases for the purpose of statistical analysis, with our first 13 cases (Coaptite I) representing the bulk of our learning curve. Doing so allowed us to limit the effect of a procedural learning curve on the results.

After controlling for the learning curve by excluding our first 13 patients, our CaHA success rates (62% Grade 0 and 75% Grade 1 success rates after two injections) were comparable to the early Dx/HA success rates reported by Läckgren et al. (59% Grade 0 and 68% Grade I success rates) [13]. The characteristics of these two groups were slightly different. Stenberg's cohort had a higher mean grade of reflux (3.15 versus 2.6) and a greater number of their patients received a second injection than in our cohort (34% versus 7%). We feel that the first 13 CaHA patients represent our learning curve, given the dramatic increase in success rates from the Coaptite I to the Coaptite II group (43% versus 62%). Therefore, the results from the Coaptite II group more closely represent the true efficacy of this procedure.

Even when controlling for the learning curve, our experience appears to show higher success rates for Dx/HA than CaHA. A possible explanation could be that our techniques required further refinement before achieving comparable results. However, a more likely explanation is that we used the STING [6] method on our CaHA cohort and the HIT [7] method for our Dx/HA cohort. The HIT method has recently been shown to result in a 10–15% improvement in outcomes which happens to the difference between our two cohorts. Therefore, accounting for the effect of the “learning curve” bias and the evolution to the HIT procedure, CaHA shows reasonable efficacy when compared to Dx/HA. Clearly, this is not conclusive evidence and further head-to-head trials using similar methods are required to show non-inferiority. However, our results indicate that it is possible that CaHA would perform reasonably well compared to Dx/HA.

In addition to the difficulty of controlling for the evolution of technique, our study may have other limitations due to its design as a retrospective case series. There was significant patient selection bias between our cohorts in regards to dysfunctional voiding. Also, we could not control for the evolution of perioperative management. Early aggressive distension of the bladder may contribute to deformation of the implant. CaHA patients received on-table cystograms moments after deployment of the biomaterial, possibly deforming the optimal 3-dimensional configuration of the implanted material. On the other hand, the final 15 patients in the Dx/HA cohort received intraoperative crushed phenoxybenzaprine (Pyridium) via nasogastric tube as well as 2% lidocaine jelly (Uro-Jet) per urethra for postoperative bladder analgesia, which likely facilitated early postoperative voiding, thereby preventing overdistension of the bladder and implant deformation/migration.

One potential weakness of this study is the relatively short follow-up period. Multiple authors have described a significant frequency of late failures regardless of whether Dx/HA or CaHA was used [11, 13–15]. Most of these studies showed failures at 1 year but not much further beyond that. Although our cohort was last injected some years ago, long-term follow up was not available for analysis. It would be interesting to see what the rate late failures are and how that corresponds to the risk of febrile urinary tract infection. However, these questions can only be answered with a prospective randomized trial with long-term follow up which is greatly needed.

We feel that CaHA may be considered a viable alternative biomaterial in the injectable treatment of VUR. Its efficacy approaches that of Dx/HA in the early reported series. The most unbiased scientific comparison of these two biomaterials in their role at correcting VUR in children will come through a prospective randomized controlled trial. In addition to controlling for technique modification, such a trial could also examine relative complication rates. Currently, there is a 0.7% reported occurrence of ureteral obstruction with Dx/HA, and case reports of periureteral fibrosis with CaHA [16–18]. In our retrospective review, no complications were observed with either material. 

## 5. Conclusions

In our single-user experience, CaHA had success rates comparable to early Dx/HA success rates. When controlling only for the learning curve, Dx/HA appeared to show higher success rates than CaHA. This may be partially explained by the addition of the intraureteric injection that was used in the Dx/HA cohort. A prospective randomized controlled trial is necessary to definitively compare the two biomaterials in their role as endoscopic treatments for VUR.

## Figures and Tables

**Table 1:** Patient demographics.

	Total CaHA	Coaptite I	Coaptite II	Dx/HA	*P* value^+^
Number of pts	27	13	14	21	N/A
Number of ureters	42	21	21	32	N/A
Male : female ratio	0.19	0.16	0.22	0.45	*P* = 0.06*
Mean age	6.8	6.8	7.1	5.0	*P* = 0.05**
Mean grade reflux	2.4	2.2	2.6	2.8	*P* = 0.02**
Patients with history of dysfunctional voiding	11	9	2	0	*P* < 0.01*

^+^Total Coaptite versus deflux.

*Fisher's Exact test.

**Student *t*-test.

**Table 2:** Distribution of VUR grade.

VUR grade	No. unilateral (%)	No. bilateral (%)*
Total patients	22	26
I	1 (4.5%)	1 (3.8%)
II	3 (13.6%)	7 (26.9%)
III	16 (72.7%)	16 (61.5%)
IV	2 (9.1%)	2 (7.7%)
V	0 (0%)	0 (0%)

*Higher grade of 2 ureters.

**Table 3:** Bioinjectable success rates for the endoscopic treatment of VUR.

Bioinjectable cohort	Single injection Grade 0* success rate	No. ureters reinjected	Multiple injection success rate	Grade I** success rate (one or two injections)
Dx/HA	25/32 (78%)	2	n/a	28/32 (88%)
CaHA (all patients)	23/42 (52%)	9	28/42 (67%)	34/42 (80%)
*P* value	*P* < 0.01			
Coaptite I	9/21 (43%)	7	13/21 (62%)	18/21 (85%)
*P* value				
(versus Dx/HA cohort)	*P* < 0.01			
Coaptite II	13/21 (62%)	2	13/21 (71%)	16/21 (75%)
*P* value				
(versus Dx/HA cohort)	*P* = 0.03			

*Grade 0 Success is defined as no reflux on postoperative VCUG.

**Grade 1 Success is defined as improvement to at least Grade 1 reflux on postoperative VCUG.
